# Understanding and Addressing the Digital Health Literacy Needs of Low-Income Limited English Proficient Asian American Patients

**DOI:** 10.1089/heq.2022.0045

**Published:** 2022-07-04

**Authors:** George Lee, Anita Chang, Agnita Pal, Thu-An Tran, Xinyue Cui, Thu Quach

**Affiliations:** Asian Health Services, Oakland, California, USA.

**Keywords:** digital health literacy, limited English proficient, remote patient monitoring, telehealth, Asian American, journey mapping

## Abstract

**Introduction:**

During the pandemic, Asian Health Services (AHS), a federally qualified health center serving patients in 14 Asian languages, transformed rapidly to provide telehealth visits, developed an intensive remote patient monitoring program, and conducted a digital health literacy survey.

**Method:**

This article describes how AHS collected and utilized descriptive data on our patient population to inform our rapid adoption of telehealth and assess our patients' response to these changes.

**Results:**

Our experiences show that audio visits are invaluable for our patients. In addition, our remote monitoring program resulted in 96% of patients improving their blood pressure control.

**Conclusion:**

Many barriers to widespread adoption of telehealth exist, including low digital literacy and the need for in-language digital training. Disaggregated data by ethnicity and language are needed to inform future work.

## Introduction

The SARS-Cov-2 (COVID-19) epidemic impacted the health care system drastically, forcing health care providers to transform care immediately from in person face-to-face visits to telehealth.^1,2^ Much of this immediate adoption meant that communities who face certain barriers, including social determinants of health factors such as language barriers and low digital health literacy, could be left behind unless there was focused attention on their needs.^3,4^

Asian Health Services (AHS), a federally qualified health center with multiple sites in the San Francisco Bay Area, serves >50,000 patients and provides >150,000 medical, dental, and mental health visits annually. Specifically, 84% of our visits are conducted in 14 different Asian languages, 92% of our patients' annual incomes are <200% of the Federal Poverty Level, and 1 in 3 patients are aged >65 years old. Our patient population, comprising older racial/ethnic minorities with limited English proficiency from low socioeconomic backgrounds, is at higher risk of experiencing digital literacy and technology access barriers.^5,6^

With the onset of the pandemic, we rapidly converted from 100% in-person visits to 90% telehealth, launched a remote patient monitoring program focused on hypertension, and initiated a digital health literacy survey of our patients.

Our research questions are as follows: (1) How did our limited English proficient (LEP) patients respond to our rapid transition to telehealth, and (2) what are our patients' level of readiness for telehealth and how does it vary across language groups?

## Methods

### Telehealth visit types

We analyzed patient utilization data from our electronic health records (EHRs) based on the different types of visits—in-person, audio, and video by patient demographics (e.g., age) to assess if there may be any trends by age category and visit type. We used chi-square tests to assess the overall statistical significance of differences across age groups. In addition, we used logistic regression to assess the statistical significance of differences between age groups taken two at a time.

### Remote monitoring

In March of 2021, AHS launched a remote patient monitoring program called Smart Hypertension Improvement Program (SHIP). This pilot program involved 29 patient participants who had uncontrolled hypertension, no evidence of a video visit, and were monolingual in Cantonese or Mandarin. We wanted to see if an intensive program involving technology solutions could be adapted successfully to a group with known language and technology barriers and in poor medical control. We provided Bluetooth-enabled blood pressure devices and iPhones with cellular data to the participants and taught them how to sync the measurements with our Epic EHR, which is designed and operated by OCHIN, a nonprofit health care innovation center.

We developed a tool that alerts us if blood pressure is in a high range or if >25% of readings are above goal. Our language concordant health coaches (i.e., trained medical assistants) called the participants weekly to biweekly to provide counseling through motivational interviewing. Critical issues were escalated to the primary care providers for management. For the analysis, we provide descriptive data on retention and outcomes. We also interviewed four patients to describe their experiences with the SHIP program.

### Digital health literacy patient survey

In response to the difficulties faced by our patients, we sought to understand their needs by conducting a digital health survey. We developed a 15-question tool and conducted usability testing with 10 Cantonese-speaking, 6 Vietnamese-speaking, and 5 English-speaking patients. We then made changes and conducted the survey with Chinese- (Cantonese/Mandarin), English-, Khmer-, Korean-, and Vietnamese-speaking patients. The questions were designed to assess their access to technology, ability to use technology, and receptiveness to using technology to communicate with their health care team. The survey study was approved by the institutional review board of the Association of Asian Pacific Community Health Organizations. For the analysis, we used descriptive statistics and performed chi-square tests to test the overall significance of differences across the language groups and logistic regression to assess the statistical significance of differences between age groups taken two at a time.

## Results

### Telehealth visit types

**FIG. 1.**
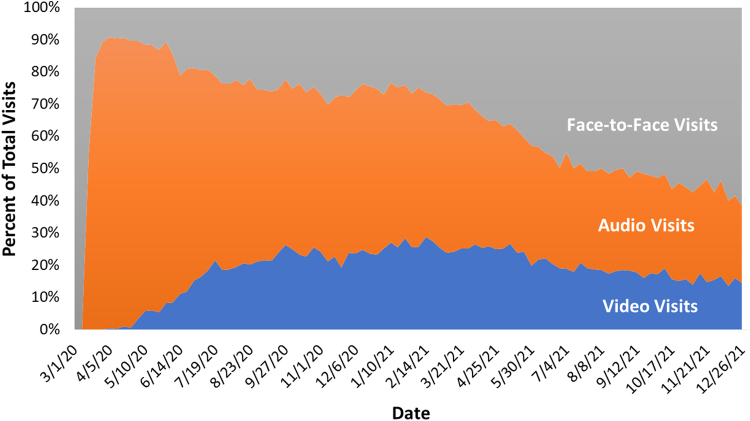
Percentage of total visits by type of visit (e.g., face-to-face, audio, and video) at Asian Health Services in Alameda County, California between March 1, 2020 and December 31, 2021.

During shelter in place, 90% of our visits were by telehealth: either by video or audio only. As pandemic restrictions relaxed over time, we settled into a steady state of about 15% video, 25% audio, and 60% in-person (Fig. 1). Based on our data of 28,147 patients who completed 178,353 medical visits from March 1, 2020 to December 31, 2021, audio visits were especially helpful for our patients aged >65 years old (Table 1). Although 97% of that age group had used some form of telehealth, 50% of them only had audio visits and relied entirely on audio to receive care. There was a statistically significant increased reliance on audio visits as age increases.

**Table 1.** Patient Utilization of Different Visit Types by Age Groups at Asian Health Services in Alameda County, California Between March 1, 2020 and December 31, 2021 (*N*=28,147)

	Ages 0–18		Ages 19–39		Ages 40–64		Ages 65+	
	Count (***n***)	%	Count (***n***)	%	Count (***n***)	%	Count (***n***)	%
Patients with only face-to-face visits^a^	1109	20	591	13	596	6.7	283	3
Patients with telehealth visit, only in audio^b^	1465	27	1932	43	4130	46.5	4700	50
Patients with at least one video visit^c^	2873	53	1979	44	4152	46.8	4337	47
Total	5447	100	4502	100	8878	100	9320	100

^a^ Chi-square test/logistic regression performed and shows highly significant difference (*p*<0.001) between age groups, taken two at a time.

^b^ Chi-square test/logistic regression performed and shows highly significant difference (*p*<0.001) between age groups, taken two at a time.

^c^ Chi-square test/logistic regression performed and shows the difference between age 0–18 and other age groups is highly significant (*p*<0.001); the difference between age 19–39 and the two oldest age groups are significant (*p*<0.05); the difference between the two oldest age groups are not significant.

### Remote monitoring

**FIG. 2.**
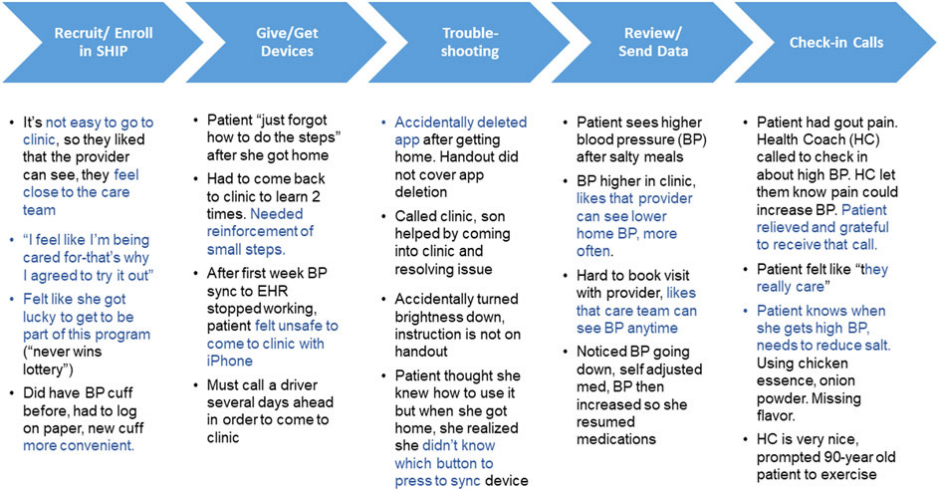
Journey Map: Patient responses regarding their experience during each stage of the Smart Hypertension Improvement Program. Compiled from four patients, ranging from ages 44 to 90 years. BP, blood pressure; EHR, electronic health record; HC, health coach; SHIP, Smart Hypertension Improvement Program.

In Table 2, our results showed that of the 23 patients retained in the program, 96% or 22 patients were in control or improved at the end of the 6-month program. In addition, 78% started to use video visits for the first time. We used journey mapping as one of our evaluation tools for patient experience (Fig. 2). Patients felt “cared for” and “close to the care team” and liked that their “PCP (primary care provider) can see the blood pressure any time.” Patients also liked seeing their blood pressure trends in apps, and developed insights into the effect of changes in their choices (e.g., for diet) on their blood pressure.

**Table 2.** Results of Smart Hypertension Improvement Program: A Pilot Intensive Program Using Technology Solutions for a Group with Known Language and Technology Barriers from March 5, 2021 to September 9, 2021 (*N*=29)

Metrics		Count (***n***)	%
1	Patients retained in the program	23	79
2	Retained patients with blood pressure in control or improved	22	96
3	Retained patients with a video visit	18	78

However, there was a clear need not only for training, which we provided but also for troubleshooting, as patients “accidentally” deleted an app or changed their settings or experienced intermittent issues with uploading blood pressure readings from remote devices to the EHR. The fear in the community from the rising Asian hate crimes^7^ was also evident in their answers, which negatively impacted their desire to come in for device troubleshooting or any health care services.

### Digital health literacy patient survey

Our preliminary findings with 759 participants (Table 3) show that overall, 66% of patients can use the internet. Most participants use the internet for either video (59%) or social media (55%) consumption. Few participants (27%) log in to an account with a user name and password. Our Chinese-, Vietnamese-, Khmer-, and Korean-speaking patients were significantly less likely to use the internet to log in. This ability to log in to an account is critical to accessing the patient portal, which is the standard tool given to patients for communication with their health care team. This low percentage calls into question the utility of a patient portal for communication in this population and underscores the need to invest in efforts to promote increased digital literacy, as well as the development of simpler user-friendly technology solutions.

**Table 3.** Preliminary Results from Asian Health Services' Digital Health Literacy Survey on Internet Usage for Patients from Five Language Groups Between January 13, 2022 and May 1, 2022 (*N*=759)

	Using internet, %^a^	Search, %	Video, %	Social media, %	Shopping, %	Login, %^b^	Banking, %
Chinese (*n*=322)	49	32	42	47	20	22	20
Vietnamese (*n*=131)	71	42	61	44	22	20	8
Korean (*n*=59)	78	71	75	78	39	34	34
Khmer (*n*=116)	72	19	72	62	16	18	14
English (*n*=131)	77	72	71	59	52	50	43
Total (*n*=759)	66	42	59	55	27	27	22

^a^ Chi-square test/logistic regression performed and shows Chinese respondents are significantly LESS likely (*p*<0.001) to use the internet than the other language groups. English, Khmer, Korean, and Vietnamese groups are NOT significantly different from each other.

^b^ Chi-square test/logistic regression performed and shows Asian language groups who use the internet for login are significantly LESS likely (*p*<0.001) than English users. There are no significant differences between Chinese, Khmer, and Vietnamese users. Korean users are significantly MORE likely (*p*<0.05) to use login than Khmer and Vietnamese.

## Conclusion

The pandemic catapulted the entire health care system into telehealth.^8^ Remote synchronous medical visits, such as video or audio visits, and asynchronous monitoring devices are now becoming the standard of care. There is a paucity of data on barriers faced by the limited English proficient Asian low-income population.^9^ Other research has discussed the digital divide in the elderly or low-income population but did not have much data on Asians.^10^ For our patients who already experience health disparities due to social inequalities, the emergence of technological innovations threatens to widen the gap even more.^11^ As the technology-enabled English-speaking population who can afford technologies progress and as systems change to meet those needs, our patients are left behind. This human toll will result in poorer health outcomes and a greater burden across our entire health care system.

Through our work, we have identified several key issues that need to be addressed to prevent this widening gap:

1. Audio visits have been a lifeline for our limited English proficient patients with 50% of those >65 years old relying on audio as the sole source of care. Afraid to come into the clinic and not having access to video technologies, patients turned to phone audio visits to receive necessary care. During the public health emergency, audio visits were allowed to be reimbursable from the California Centers for Medicare and Medicaid Services.^12^ However, this poses financial risks for our organization once the public health emergency is lifted and we are no longer reimbursed for audio visits. We should advocate for audio visits to be reimbursable on an ongoing basis. Otherwise, underserved populations who face language and socioeconomic barriers, along with those who provide care for them will be disproportionately impacted.
2. Our interviews and digital health survey show that patients need our help to learn how to use video visits, remote monitoring devices, and other communication tools. Troubleshooting, not just teaching, is both essential and time-consuming. Support that is provided in language is crucial. Developing a system of technical assistance throughout the community (such as libraries, community health centers, fairs, senior centers, and churches) may provide a possible solution.
3. Remote patient monitoring works. However, the heavy use of staff time to teach and troubleshoot the technology and the difficulty in getting data from the device to the EHR show that significant improvements are needed to make it sustainable and scalable for our population. We should develop partnerships between patients, health care staff, technology companies, insurers, and EHR companies to co-create apps that integrate seamlessly across platforms, allow the care team easy access to actionable data and promote patient engagement in their own language.
4. To be effective, we need to support patients where they are. The level of support will vary, but the first step is understanding our patients' needs. Our preliminary survey results show the differences between the different Asian populations in their use of technology. Having data that establish the needs of specific communities will be critical to reaching them. We should encourage more collection of data in a disaggregated way.

This has been a transformative time in health care. Necessity has forced innovation, but the reality is that those who are most vulnerable run the risk of being left further behind. Unless we act, they will be left with a standard of care that is a relic from a decade ago.

## Abbreviations Used

AHS: Asian Health Services
BP: blood pressure
EHRs: electronic health records
HC: health coach
LEP: limited English proficient
SHIP: Smart Hypertension Improvement Program
